# Effects of Different Low-Intensity Exercise Types on Duration, Energy Expenditure and Perceived Exertion in Obese Individuals

**DOI:** 10.3390/ijerph19084893

**Published:** 2022-04-18

**Authors:** Mohamed Ali Khanfir, Hassen Ben Awicha, Liwa Masmoudi, Faten Ben Hmadou, Wajdi Dardouri, Sultan Alardan, Sabeur Nouira, Mohamed Zouch

**Affiliations:** 1 Department of Sport Sciences and Physical Activity, College of Education, University of Hail, Hail 81411, Saudi Arabia; wajdi.dardouri@gmail.com (W.D.); sultan-alardan@hotmail.com (S.A.); mohamedzouch@yahoo.fr (M.Z.); 2 Research Laboratory: Education, Motricity, Sport and Health, EM2S, LR19JS01, High Institute of Sport and Physical Education of Sfax, University of Sfax, Sfax 3000, Tunisia; hassenawicha@hotmail.fr (H.B.A.); liwa.masmoudi@yahoo.fr (L.M.); sabernouira@gmail.com (S.N.); 3 Sectorial Center of Medicine and Sports Sciences, Sfax 3000, Tunisia; abidfaten@ymail.com; 4 Research Laboratory of Exercise Physiology and Pathophysiology: From Integral to Molecular “Biology, Medicine and Health” (LR19ES09), Faculty of Medicine of Sousse, University of Sousse, Sousse 4054, Tunisia

**Keywords:** obesity, fat max intensity, exhaustive exercise sessions, RPE, energy expenditure, fat oxidation rate

## Abstract

Physical exercise is a common strategy in overweight and obesity management. Exercise type, intensity, duration, energy expenditure and the rate of perceived exertion (RPE) are the essential determinants of exercise efficiency. The purpose of the present study was to compare continuous and intermittent exercises targeted at the maximal fat oxidation intensity (FAT max) in obese individuals. Ten obese males (BMI > 30 kg/m^2^; age: 19 to 35 years) who maintained a sedentary lifestyle were recruited for this study to perform three separate exhaustive exercises: a continuous exercise at FAT max (CON), an intermittent exercise that alternates two minutes at FAT max −10% with one minute at FAT max +20% (INT½), and a second intermittent exercise that alternates four minutes at FAT max −10% with one minute at FAT max +40% (INT¼). The duration of the INT¼ exercise (65.1 min ± 13.4) was significantly longer than that of the CON exercise (55.4 min ± 6.0). No significant difference in the total amount of energy expenditure was observed across the three types of exercise (CON: 372 Kcal ± 98.2, INT¼: 398 Kcal ± 145.5, INT½: 374.4 Kcal ± 116.1). The fat oxidation rate after 45 min during the INT exercises (INT¼: 93.0 ± 19.1 mg/min, INT½: 71.1 ± 15.6 mg/min) was significantly higher than that of the CON exercise (36.1 ± 12.2 mg/min). The CON exercise was less well tolerated. The rate of perceived exertion (RPE) at the end of the CON (15.8 ± 2) was significantly higher than that of the INT exercises (13.5 ± 2 for the INT¼ and 13.1 ± 1.8 for the INT½). The INT exercises were more efficient in terms of duration, fat oxidation and RPE.

## 1. Introduction

Obesity is a complex condition that, in simple terms, is caused by a chronic imbalance between energy intake and energy expenditure [1]. According to World Health Organization data, 1.9 billion people worldwide are overweight, with 650 million of those considered obese [2]. Noncommunicable diseases, which are associated with obesity, are the leading cause of death worldwide [3]. Obesity is linked to an increased risk of developing a variety of diseases, such as type 2 diabetes mellitus (T2DM), coronary heart disease, stroke and certain types of cancer [4,5]. Physical activity is a key determinant of energy expenditure, and is thus fundamental to energy balance and weight control [3]. Due to the significant rise in energy expenditure associated with it, physical exercise is widely credited as an effective intervention for weight loss [6]. Data from the literature support the hypothesis that weight loss and weight loss maintenance are regulated by the total energy expenditure rather than exercise intensity [7,8]. Exercise prescriptions for obese individuals define the type, intensity, duration, and frequency of the exercise [9].

Intensity and duration must be manipulated so that the intensity is low enough to allow a suitable duration to expend the recommended caloric energy. The American College of Sports Medicine (ACSM) recommends a training frequency of five to seven weekly sessions of 45 to 60 min of moderate intensity, due to deconditioning, and emphasizes frequency and duration rather than intensity [10]. The volume of exercise needed for weight loss is greater than that which is necessary to improve fitness [11]. In this regard, 200–300 min of physical activity per week has been suggested for long-term weight loss and prevention of weight regaining [12]. According to the American National Weight Control Registry (NWCR), successful weight maintainers reported higher levels of physical activity (on average 2800 kcal/wk equal to 60–90 min/day with moderate intensity) than the traditionally recommended physical activity target (1000 kcal/wk) for weight control [13]. A decrease in physical activity below this target is a predictor of weight regain over time [13,14]. Significant weight loss is possible with aerobic exercise training (ET) without caloric restriction, but it requires a high ET volume. For the general population, these ET volumes may not be practical or sustainable [15]. Some authors support that the duration of daily physical activity should not be restricted unless signs of suffering from the effects of excessive activity are observed [16]. Jakicic (2015) reported that in spite of physical activity being predictive of improved long-term weight loss and minimized weight regain [7,17,18], the maintenance of a sufficient dose of physical activity is challenging, and adherence is typically below the optimal level, which could impact long-term weight outcomes [19].

To enhance exercise training adherence, the choice of exercise training type and intensity is important [16]. Exercise intensity is a crucial component of exercise prescription that may affect exercise adherence: exercise intensity may be related to how tolerable participants perceive the exercise to be, and higher exercise intensities have been shown to be associated with reduced exercise adherence [20]. Along with heart rate (HR), the rating of perceived exertion (RPE) provides a useful tool to gauge exercise intensity [10]. The consensus concerning intensity is that moderate-intensity exercise at 55% to 69% of maximum heart rate is appropriate for the management of body weight, rather than activity of more vigorous intensity [9]. Multiple studies have shown that fatty acid handling and oxidation are impaired in the skeletal muscles of obese, impaired glucose-tolerant, and T2D individuals [21,22,23,24,25]; this defect leads to proposed exercise protocols that aim at restoring muscular ability to oxidize lipids. For this reason, Low Intensity (LI) protocols designed for oxidizing more lipids during exercise sessions were described by several authors [21,26] and were shown to improve both the ability to oxidize lipids and body composition [27]. The low intensity that elicits maximal fat oxidation (FAT max) during a graded exercise test has been suggested as a reference method to prescribe exercise training where optimizing fat oxidation is the goal [28,29]. A meta-analysis of 12 FAT max training studies showed that three to four weekly sessions of 45 min of cycling at the FAT max resulted in a weight loss of −2.25% (confidence range −3.53 to −0.97), which is at least as efficient as the various protocols studied in the literature [30]. As to exercise type, whereas Venables (2008) maintains that a continuous exercise training protocol elicits high rates of fat oxidation, a high contribution of fat to substrate oxidation during exercise and better insulin sensitivity compared with a eucaloric interval protocol [31], Leanne Campbell (2010) showed that interval, as opposed to continuous, exercise training has similar effects on body mass, fat mass, lean mass and adherence to exercise [32]. Moreover, Coquart (2009) indicated that intermittent exercise is perceived as less hard by obese patients and has beneficial effects on body mass, heart rate and performance [33].

Therefore, the purpose of the present study is to compare continuous and intermittent exercises targeted at FAT max intensity in obese individuals in terms of total energy expenditure, substrate utilization and perceived exertion.

## 2. Materials and Methods

### 2.1. Participants

The a priori sample size was calculated using G*Power software (version 3.1.9.4; Kiel University, Kiel, Germany) [34]. Values for α were set at 0.05 and power at 0.9. The minimum effect size used for the calculation of sample size was based on the study of Mazurek (2016), who reported an effect size ranging from 0.51 to 0.55 for parameters close to ours [35]. Accordingly, the authors opted for an effect size of 0.51. Therefore, the required power may be obtained with the data of at least 10 participants.

Ten obese males (BMI > 30 kg/m^2^; age: 19 to 35 years) who maintained a sedentary lifestyle were recruited for this study. All participants were nonsmokers, not diagnosed with diabetes, hypertension, and/or coronary heart disease, on no medication known to influence metabolic responses and had no strenuous exercise on the day preceding any of the experimentations. They received detailed information on the study prior to the baseline test and gave their informed consent.

The study was conducted according to the declaration of Helsinki and was approved by the Research Ethics Committee (CPP n°/023/2012).

### 2.2. Study Design

All subjects participated in 4 exhaustive exercise sessions with intervals of 3 to 5 days. The first session consisted in a diagnostic incremental exercise test (IET) to determine the FAT max and VO_2_ max of each participant. The remaining sessions simulated 3 low-intensity exercise training types targeted close to FAT max intensity: a continuous exercise at FAT max (CON), an intermittent exercise that alternated two minutes at FAT max −10% with one minute at FAT max +20% (INT½), and a second intermittent exercise that alternated four minutes at FAT max −10% with one minute at FAT max +40% (INT¼). The order of exhaustive exercises was randomized and followed a counterbalanced format.

### 2.3. Testing Protocol

During a preliminary session, all patients reported their physical activity and current medication, if any. Next, they were familiarized with the RPE scale of Borg [36] and handed the instruction sheet. This scale comprises 15 numerical ratings (between 6 and 20) associated with verbal cues, from ‘‘7 = very very light’’ to ‘‘19 = very very hard’’. Each participant was asked, ‘‘How hard do you feel this exercise is?’’

All exercises were conducted on an electromagnetic cycle ergometer (Ergometrics 800, Ergoline^®^, Bitz, Germany). Respiratory gas analysis was carried out throughout the exercises via a breath-by-breath system with an open-circuit gas analysis system (ZAN 600 WHOLE SYSTEM TB600/005).

The IET [29] began with an initial 3-min warm-up period at 20% of the Predicted Maximum Power (PMP), followed by four 6-min steps at 30, 40, 50 and 60% of PMP, respectively. Oxygen uptake (VO_2_) and carbon dioxide production (VCO_2_) were averaged over the last two minutes of each step. The 6-min steps were more suitable for very sedentary patients than the 3-min ones [37]. After the last 6-min step, two to three 1-min steps were performed to meet the classical criteria of maximality of the test. The recovery phase included two periods during which respiratory and cardiac parameters were monitored: active recovery at 20% of the PMP in the first minute and passive recovery in the next two minutes. In the last two minutes of each step, the values of VO_2_ and VCO_2_ were recorded to calculate the respective rates of carbohydrates and fat oxidation using the classical stoichiometric equations of indirect calorimetry [38]:

Carbohydrates (mg/min) = 4.585 VCO_2_ − 3.2255 VO_2_ (1)

Fat Oxidation (mg/min) = −1.7012 VCO_2_ + 1.6946 VO_2_ (2)

The exercise intensity at which the highest rate of fat oxidation occurred was defined as the FAT max intensity and was recorded individually, to be applied in the simulation continuous and intermittent exercise training sessions.

For more accurate RPE values corresponding to the simulation exercise training sessions, an anchoring procedure was proposed in the IET. Indeed, participants were instructed to assign the lowest numerical rating on the RPE scale to their feeling of exertion when they are at rest prior to warm-up, and the highest rating at the maximal aerobic power at the end of the test.

During the exhaustive continuous (CON) and intermittent (INT) exercise sessions, the VO_2_ and VCO_2_ values were recorded during the last 15 s of each minute to calculate the respective rates of oxidation of carbohydrates and fats. RPE values corresponding to an overall perception of exertion were also collected during the last 15 s of each minute.

### 2.4. Statistical Analysis

Statistical data analysis was performed using STATISTICA software (StatSoft, version 12, Paris, France). Data in the text and in the tables are presented as means ± standard deviations. In Figures, individual data and Box plots values: the minimum value, the first quartile, the median, the third quartile, and the maximum value are reported. The Shapiro Wilk test was applied to check the normality of distributions. To compare the three exercise types, “Exercise durations”, “Total energy” and “Fat energy after 45 min” were analyzed using a one-way repeated measures analysis of variance (ANOVA). “Fat oxidation” and “RPE” were analyzed using a two-way repeated measures ANOVA (3 “Exercise types” (CON, INT½ and INT¼) × 2 “Exercise durations” (Before 45 min vs. After 45 min)). When appropriate, a Scheffé post hoc analysis test was conducted to compare parameters in pairs. Effect sizes were calculated as partial eta-squared (η_p_^2^) for ANOVA (values of η_p_^2^ ≥ 0.01 indicated small, ≥0.06 medium and ≥0.14 large effect sizes, respectively), and as Cohen’s d to compare each pair of the three exercise modes (values of d ≥ 0.2 indicated small, d ≥ 0.5 medium and d ≥ 0.8 large effect sizes, respectively). All statistics were considered significant at a probability threshold of 5% (*p* < 0.05).

## 3. Results

Anthropometric data and physiological values measured during the exhaustive incremental exercise are presented in Table 1.

The variation of the exercise types induced different exercise durations but similar energy expenditures (Table 2). The participants performed the three exercise types close to FAT max intensity for a duration exceeding 45 min, i.e., the average duration often prescribed for obese patients [39]. The longest duration was recorded in the INT ¼ exercise. The duration of the INT ¼ exercise was significantly longer compared to the CON exercise.

In spite of the low exercise intensity, the long duration fixed by the time limit allowed a relatively substantial expenditure of energy close to 400 kcal per session for the three types of exercise. No significant difference in the total amount of energy expended was observed across the three types of exercise, despite the significantly longer duration of the INT¼ exercise compared to the CON exercise (Table 2).

Concerning fat oxidation, the statistics showed a significant interaction between exercise type × exercise duration (F_(2,18)_ = 8.05; *p* = 0.003; η_p_^2^ = 0.413). The energy expenditure from fat after 45 min in both of the INT exercises was significantly higher compared to the CON exercise (Table 2).

**Figure 1:**
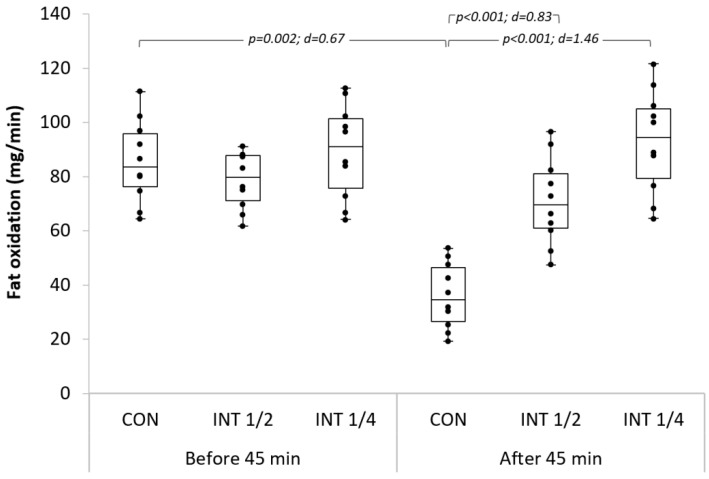
Fat oxidation rates of the three exercise types before and after 45 min; d: Cohen’s d.

The fat oxidation rate after 45 min of work decreased significantly in the CON exercise, while it remained constant for both of the INT exercises. After 45 min, in the INT½ and INT¼ exercises, the fat oxidation rate was significantly higher than in the CON exercise. No difference was observed between INT½ and INT¼ concerning the fat oxidation rate above 45 min. (Figure 1).

**Figure 2:**
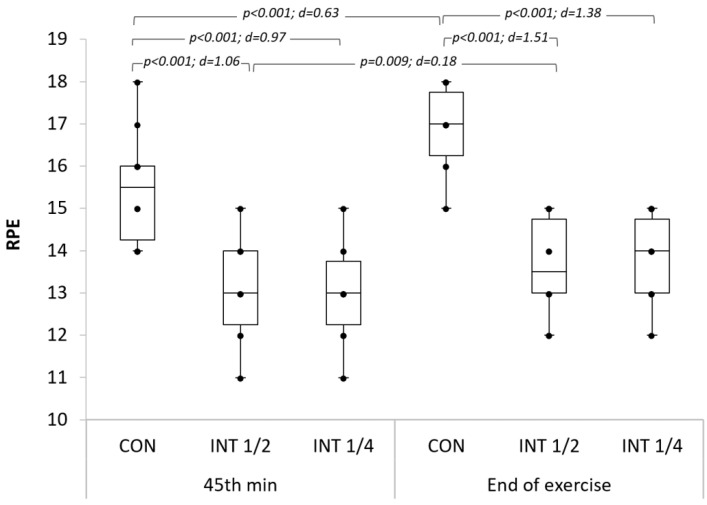
RPE at the 45th minute and at the end of the three exercise types; d: Cohen’s d.

The RPE statistics showed a significant interaction between exercise type × exercise duration (F_(2,18)_ = 9.31; *p* = 0.002; η_p_^2^ = 0.485). The results of the perceived exertion using the Borg Scale showed that the CON exercise is less well tolerated than the INT exercises. At the 45th minute and at the end of exercise, the perceived exertion values in the CON exercise were significantly higher than those in the INT exercises. Unlike the CON exercise and the INT½ exercise, the difference in perceived exertion between the 45th minute and the end of the INT¼ exercise was not significant (Figure 2).

## 4. Discussion

The aim of the present study was to compare in obese individuals the efficiency of different types of individualized exercises targeted at FAT max intensity in terms of exercise duration, total energy expenditure, fat oxidation and perceived exertion.

Standard fitness exercise prescriptions for obese individuals focus on greater overall energy expenditure with a modest intensity, on account of deconditioning, and emphasize duration and frequency rather than intensity [9]. With a low-intensity exercise fixed close to FAT max, we tried to measure the maximum duration that an obese individual can endure for different types of exercise. According to Donnelley (2009), for long-term weight loss and for the prevention of weight regain in obese individuals, exercise duration should reach 250–300 min per week [40]. The ACSM recommends exercise programs exceeding 225 min per week in order to possibly induce clinically significant weight loss [15]. The CON and INT exercises proposed in this study can easily reach this target with four or five sessions a week. In the CON exercise as well as the INT exercises, the participants in this study exceeded the duration of 45 min frequently recommended for obesity. They cycled for more than one hour in the INT exercises (62 min for the INT½ and 65 min for the INT¼) and 55 min in the CON exercise. Statistically, only the INT¼ exercise was significantly longer than the CON exercise. It seems that monotony accounts for the relatively shorter duration of the CON exercise. A small variation in intensity during the INT½ exercise showed a tendency to increase the duration, though not significantly. A more marked variation in intensity during the INT¼ exercise made it possible to observe a significant increase in the duration of the effort. With an effort duration of more than 60 min from the first training session, the proposed INT exercises fall within the duration ranges recommended for obese people by the American NWCR: 60 to 90 min/day [16], and the American Institute of Medicine: at least 60 min/day of exercise on most weekdays [41].

To improve long-term weight loss, exercising for 250 to 300 min per week (or ~60 min per day), which is equivalent to about 2000 kcal energy expenditure per week, is commonly recommended. For more efficiency in weight loss, a target expenditure of 2000 to 2800 kcal per week is reported [16]. With a total energy expenditure close to 400 kcal per session measured in the three types of exercise that we proposed, five to six sessions per week can easily reach the amount of energy expenditure recommended for weight loss and weight-loss maintenance.

Despite the relatively longer exercise duration noted during the INT exercises, energy expenditure during the CON exercise was as high as the INT exercises, and no significant difference was observed. The extra energy expended during the CON exercise that made up the difference in working time with the INT exercises could be explained by the parasitic movements typically seen with fatigue. In longer exercises, the arising fatigue could be attributed to an alteration in the energy efficiency linked to the peripheral effectors, which can alter the energy cost [42]. Psychogenic factors, such as cognition, perception and affect, have also been identified as factors that may influence the mechanical cost [43]: the RPE at the end of the effort was significantly higher in the CON exercise compared to the INT exercises.

Few studies refer to the substrate (fat or CHO) that is oxidized during exercise. However, multiple studies have shown that fatty acid handling and oxidation is impaired in the skeletal muscles of obese, impaired glucose-tolerant, and T2D individuals [21,22,23,24,25]. This defect leads to proposed exercise protocols that aim at restoring muscular ability to oxidize lipids. Low-intensity protocols, such as FAT max intensity, are designed for oxidizing more lipids during exercise sessions [21,26] and are shown to improve both the ability to oxidize lipids and body composition [27].

Although continuous exercise is shown as more efficient in increasing fat oxidation after training [31], in our study, we noted that, (i) the fat oxidation rate decreased significantly after 45 min during the CON exercise, while it remained constant for both of the INT exercises, and (ii) the energy from fat oxidation after 45 min during the INT exercises was significantly higher than that in the CON exercise. As shown before, the participants in this study finished the CON training session in a less comfortable state compared to the INT ones. This may explain a drift towards the use of carbohydrates at the end of the CON exercise. Longer-duration training at a lower intensity is known to use glycogen as a predominant energy source during the first 20 min of exercise, followed by a shift to fat stores after 30 min [11]. We noted that, above 45 min of low-intensity CON exercise, this shift may reverse due to the efficiency of the energy yield from carbohydrates. Mark Hargreaves (2020) explained that, in endurance exercise, energy use shifts to carbohydrates, and reliance on fat decreases, because the energy yield from carbohydrates for aerobic ATP production is more efficient than that from fat [44]. The INT exercises we proposed have shown more efficiency in the mobilization of fats beyond 45 min of exercise.

In this study, the RPE, measured at the 45th minute as well as at the end of the exercises, showed that the INT types of work were better tolerated by participants than the CON type: the CON exercise was rated “hard” at the 45th minute and “very hard” at the end of the exercise, while the INT exercises were perceived as “somewhat hard” at these same exercise moments. These levels of perceived exertion further support our explanation for the decrease in fat utilization at the end of the CON exercise compared to the INT exercises.

Despite the positive effect on health, obese individuals have high dropout rates in exercise programs [45]. The main reason is linked to the negative experience in the practice of physical exercise associated with high ratings of perceived exertion [46]. In order to “allow adherence to be sufficient for desired biological changes to occur”, Dishman (1982) recognized the need to find a “compromise” between the “ideal physiological prescription” and a “manageable behavioral prescription” [47]. To promote adherence, the 8th edition of the Guidelines for Exercise Testing and Prescription of the ACSM recommended the RPE (ratings of perceived exertion) in prescribing exercise intensity. For obesity, the initial aerobic exercise training intensity should be moderate: 40–60% VO_2_ max or HRR, and perceived as somewhat hard: 12 to 13 on the 6–20 Borg Scale [48]. By maintaining a low rating of perceived exertion (RPE = 13) above 60 min of exercise, the INT exercises we proposed, and especially the INT¼, are within the intensity range recommended by the ACSM to increase the adherence levels and decrease the dropout rates of obese individuals. Therefore, it can be an effective alternative in the training prescription for obesity.

Some limitations of this study include the small number of participants. First, though required statistical power is met with 10 experimental subjects, we had to calculate the effect size of each parameter. Moreover, the small number of participants allowed for the investigation of physical exercise responses in individuals with a BMI > 30. Yet, the responses to physical exercise, namely the fat oxidation rate and RPE, may vary according to obesity classes. Further investigation should examine responses to the low-intensity exercises we proposed across obesity classes I, II and III. Finally, the study results were based on acute exercises only, and they should be verified by long-term training programs. The major finding of this research lies in limiting long-term training programs to intermittent exercises.

## 5. Conclusions

In conclusion, the INT exercises fixed close to FAT max intensity in obese individuals allowed for a longer exercise duration (over 60 min), more fat oxidation above 45 min of exercise and a lower rating of perceived exertion than the CON exercise fixed at FAT max intensity. The applied INT exercises were effective in terms of duration, energy expenditure, fat oxidation and RPE. When physical exercise in obese individuals seeks to increase energy expenditure, fat oxidation and adherence, the INT exercises we used can be a real alternative in exercise prescription. Thus, longitudinal studies should verify the effect of this personalized training on reducing body weight and fat mass, maintaining weight loss and adherence, and changing lifestyle and health in obese individuals.

## Figures and Tables

**Table 1:** Anthropometric data and physiological values measured during the exhaustive incremental exercise (*n* = 10).

Characteristics	Mean ± SD	[Range]
Age (years)	26.1 ± 6.0	[19; 35]
Body weight (kg)	104.2 ± 19,4	[87; 150]
Height (cm)	175.7 ± 8.8	[166; 190]
Body mass index (kg/m^2^)	33.5 ± 3.6	[30.1; 41.6]
VO_2_ peak (ml/min/kg)	30 ± 8.9	[16.2; 46.6]
Maximal fat oxidation rate (mg/min)	133.8 ± 33.4	[88.7; 220.4]
% VO_2_ peak at FAT max	30.3 ± 5.3	[24; 37]

**Table 2:** Duration and energy expenditure of the three exercise types.

Variables	Mean ± SD			ANOVA		
	Continuous	Intermittent ½	Intermittent ¼	F_(2,18)_	*p*-Value	η_p_^2^
Exercise duration (min)	55.4 ± 6.0	61.6 ± 6.6	65.1 ± 13.4 * (*p* = 0.017; d = 0.75)	5.23	0.016	0.368
Total energy (kcal)	372 ± 98.2	374.4 ± 116.1	398 ± 145.5	0.58	0.569	0.060
Fat energy after 45 min (kcal)	3.9 ± 3.8	14.4 ± 13.2 * (*p* = 0.045; d = 0.72)	16.2 ± 16.5 * (*p* = 0.029; d = 0.73)	5.09	0.018	0.361

*: Significantly different from continuous exercise; d: Cohen’s d.

## Data Availability

The data that support the findings of this study are available on request from the first author, Mohamed Ali Khanfir.
